# The Structure of Bilirubin Oxidase from *Bacillus pumilus* Reveals a Unique Disulfide Bond for Site-Specific Direct Electron Transfer

**DOI:** 10.3390/bios12050258

**Published:** 2022-04-19

**Authors:** Shalev Gihaz, Nidaa Shrara Herzallh, Yifat Cohen, Oren Bachar, Ayelet Fishman, Omer Yehezkeli

**Affiliations:** 1 Department of Biotechnology and Food Engineering, Technion—Israel Institute of Technology, Haifa 3200003, Israel; shalevgihaz@gmail.com (S.G.); nidaa@campus.technion.ac.il (N.S.H.); yifat@bfe.technion.ac.il (Y.C.); orenchus@campus.technion.ac.il (O.B.); 2 Russell Berrie Nanotechnology Institute, Technion—Israel Institute of Technology, Haifa 3200003, Israel; 3 The Nancy and Stephen Grand Technion Energy Program, Technion—Israel Institute of Technology, Haifa 3200003, Israel

**Keywords:** Bilirubin oxidase, *Bacillus pumilus*, bioelectrocatalysis, X-ray crystallography, electron transfer, site-specific immobilization

## Abstract

Efficient oxygen-reducing biocatalysts are essential for the development of biofuel cells or photo-bioelectrochemical applications. Bilirubin oxidase (BOD) is a promising biocatalyst for oxygen reduction processes at neutral pH and low overpotentials. BOD has been extensively investigated over the last few decades. While the enzyme’s internal electron transfer process and methods to establish electrical communication with electrodes have been elucidated, a crystal structure of BOD from bacterial origin has never been determined. Here we present the first crystal structure of BOD from *Bacillus pumilus* (*Bp*BOD) at 3.5 Å resolution. Overall, *Bp*BOD shows high homology with the fungal enzymes; however, it holds a unique surface-exposed disulfide bond between Cys229 and Cys322 residues. We present methodologies to orient the T1 site towards the electrode by coupling the reduced disulfide bond with maleimide moiety on the electrodes. The developed configurations were further investigated and revealed improved direct electron transfer rates with the electrodes. The work presented here may contribute to the construction of rationally designed bioanodes or biocathode configurations that are based on redox-active enzymes.

## 1. Introduction

Multicopper enzymes have drawn increasing attention over the last few decades [1,2]. Among other functions, these enzymes have a crucial role in the degradation or oxidation processes of many metabolites both in eukaryotes and prokaryotes. While some mechanistic questions are not fully answered yet, it has been determined that multicopper enzyme active sites form high oxidation potentials that enable versatile chemical reactions [3]. These enzymes are frequently used in applied biocatalysis or sensing. For example, methane monooxygenase consists of two copper ions in its active center facilitating the conversion of methane into methanol [4,5]. Tyrosinase, a type-3 copper enzyme that initiates the formation of melanin in numerous organisms, can also be harnessed for detoxification of phenol-containing wastewater or for the production of L-dihydroxy-phenyl-alanine (L-DOPA) [6], an important drug [7]. By establishing electrical communication between multicopper enzymes and electrodes, bioelectrocatalysis or biofuel cell applications can be developed [8,9].

Bilirubin oxidase (BOD) is another well-studied multicopper enzyme. It contains four copper ions and allows the oxidation of bilirubin as part of the heme metabolic cycle [10]. It has been shown that when coupled with electrodes, the BOD enzyme can be utilized for oxygen reduction reactions with minimal overpotentials [11,12]. While many redox-active enzymes are electronically isolated from their surroundings, BOD can be activated by a direct electron transfer mechanism that occurs through its T1 site. The latter acts as an internal redox mediator and by a sequential electron transfer process activates the T2/T3 site. In turn, the active site enables the reduction process of atmospheric oxygen into water [13,14]. BOD was previously isolated from fungi or bacteria and exhibited excellent oxygen reduction abilities that were exploited for the construction of biocathodes and utilized in biofuel cells or photo-bioelectrochemical cell applications [11,12,15,16]. Over the last decades, successful attempts to elucidate the enzyme structure and mechanism have been carried out [3,8,17,18]. The internal electron transfer process from the T1 toward the T2/T3 sites was determined, and the oxygen reduction process was clarified [17,18]. However, all of the obtained data were based on fungal BOD structures. A decade ago, a bacterial multicopper enzyme originating from *Bacillus pumilus* was successfully cloned into *E. coli* [19]. Mano et al. later identified, classified, and studied the enzyme electrochemically and spectroscopically, concluding that the isolated *B. pumilus* enzyme should be classified as a bilirubin oxidase (*Bp*BOD), and not as a laccase [14,17].

Efficient oxygen reduction catalysts are required for oxygenic fuel cells. While novel metals such as Pt can facilitate four-electron O_2_ reduction processes efficiently, the use of rare metals is not sustainable, and therefore, alternatives should be explored. Moreover, at neutral pH, BOD requires a lower overpotential for its activation as compared to a Pt electrode [20]. Industrial applications of biofuel cell devices are currently limited due to stability issues; however, the activation of biomedical devices using an oxygen reduction biocathode coupled with lactate or glucose oxidizing bioanodes is feasible and therefore should be pursued. Such BOD-based cells were extensively studied leading to an improved understanding of the internal electron transfer process between the T1, T2/T3, and oxygen-binding sites [18,21]. The fungal BOD crystal structures revealed a hydrophobic pocket in close proximity to the T1 site that enabled hydrophobic interactions and direct electron transfer (DET) processes [22,23]. Anthracene [24], pyrene [25], porphyrin [26], single-walled carbon nanotubes [27,28], and multi-walled carbon nanotubes (MWCNTs) [29] were successfully tested to show the process of direct electron transfer to the T1 site. Mediated electron transfer (MET) configurations have been developed for BOD activation as well. Os-based polymers with tailored redox potential or 2,2′-azino-bis(3-ethylbenzothiazoline-6-sulfonic acid) (ABTS) redox mediator and derivatives [30] were utilized for short distance electron transfer processes [31].

**Figure 1:**
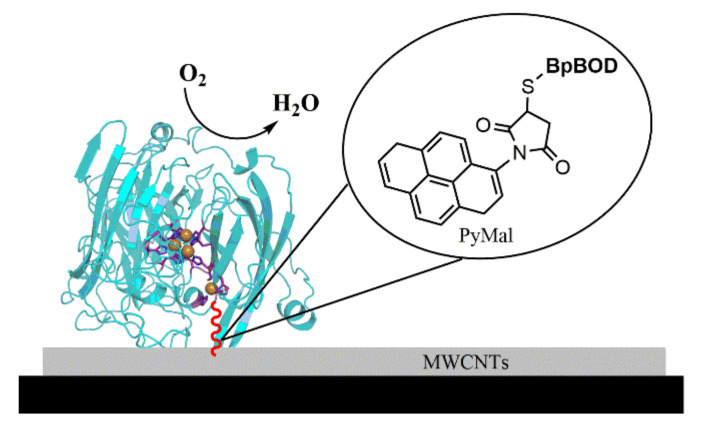
A schematic diagram of the *Bp*BOD-based electrode. The glassy carbon electrode was modified with MWCNTs, followed by a modification with PyMal (shown in red, not in scale) and a mixture containing the reduced *Bp*BOD (PDB: 7Z5P).

To date, only a few crystal structures of BOD have been determined, all from fungal species such as *Magnaporthae oryzae* [3] or *Myrothecium verrucaria* [32,33,34,35]. These solved structures enabled important progress toward solving the BOD activation mechanism and the development of bioelectrocatalysis applications [3,32,33,34,35]. Rationally designed variants may lead to further progress with optimized configurations that generate higher bioelectrocatalyitic currents. Here, we report the first crystal structure of the bacterial *Bp*BOD solved at 3.5 Å resolution. The structure revealed a unique disulfide bond in the vicinity of the T1 electron acceptor site. We further present methods to conjugate the reduced disulfide bond with a pyrene-maleimide (PyMal) linker in a site-specific fashion. The optimized hybrid dictates an improved orientation toward the electrodes, which is translated to an enhanced bioelectrocatalytic current (Figure 1).

## 2. Materials and Methods

### 2.1. Materials

MWCNTs were purchased from Nanointegris. Glassy carbon electrodes (GCEs, 3 mm diameter) were purchased from CH-Instruments. ABTS, N-(1-Pyrenyl)maleimide (PyMal), and 2-Mercaptoethanol was purchased from Sigma-Aldrich (St. Louis, MO, USA). Tris(2-carboxyethyl)phosphine) (TCEP) was purchased from Alfa Aesar. Dimethylformamide (DMF) was purchased from Bio-lab.

### 2.2. Cloning, Expression, and Purification of BpBOD

Recombinant *Bacillus pumilus* bilirubin oxidase (*Bp*BOD, UniProt A8FAG9) fused to a His-tag was expressed in *E. coli* BL21 (NEB, USA) after cloning into a pET21a vector. The cells were grown in Luria-Bertani (LB) medium and the over-expressed enzyme was purified. Cells harboring pET21a/*Bp*BOD plasmid were cultured overnight in 50 mL LB medium supplemented with ampicillin (100 μg/mL) at 37 °C and 180 rpm. The following day, 2 L shake flasks containing 0.5 L LB medium supplemented with ampicillin were inoculated with 5 mL from the preculture and incubated at 37 °C and 180 rpm. When the culture OD_600_ reached ~0.6, *Bp*BOD expression was induced at 20 °C by the addition of 1 mM IPTG and 0.5 mM CuSO_4_. Following 4 h of induction, the 0.5 L culture was poured into a 0.5 L flask and the agitation was switched off to achieve micro-anaerobic conditions. After 20 h of static-conditions inoculation, the cells were harvested by centrifugation at 8000× *g* for 10 min. Cells harboring pET21a plasmid only served as control cells and were inoculated the same way. The cells were suspended in binding buffer (50 mM Tris HCl pH 7.5, 500 mM NaCl, 20 mM imidazole) and lysed using a homogenizer (EmulsiFlex-C3 High-Pressure Homogenizer, AVESTIN, Ottawa, ON, Canada). Following centrifugation at 16,000× *g* for 20 min, clarified cell extract was obtained and used for affinity purification with Ni-NTA column in AKTA Prime Plus instrument (GE Healthcare Bio-Sciences AB, Uppsala, Sweden). The enzyme was eluted using elution buffer (50 mM Tris HCl pH 7.5, 500 mM NaCl, 500 mM imidazole) in gradient or stepwise mode. Samples were analyzed using SDS-PAGE to verify their purity. In some cases, an additional purification step using size exclusion chromatography was applied. In those cases, affinity-based fractions eluted with 260 mM imidazole were combined, concentrated, and used for SEC. SDS-PAGE gels and representative chromatograms are shown in Figure S1. Elution fractions were dialyzed against BOD buffer (50 mM Tris HCl pH 7.5, 100 mM NaCl) and BOD activity was tested with the addition of ABTS solution and the formation of a turquoise pigment, compared to the control (buffer with no enzyme) as presented in Figure S1.

### 2.3. Crystallization, Data Collection, and Structure Determination

*Bp*BOD crystallization attempts were performed in 96-well hanging and sitting drop plates using a MOSQUITO robot with the following screen suites: Crystal Screen HT™ (Hampton research, Aliso Viejo, CA, USA), PEG/Ion HT™ (Hampton research, USA), JCSG (Molecular Dimensions), and Index (Hampton research, Aliso Viejo, CA, USA) at 20 °C. The purified *Bp*BOD was screened for crystallization at a wide concentration range from 5.5 up to 20 mg/mL. Bluish crystals appeared after a few months while optimal and uniform crystals were formed at 0.1 M Sodium citrate pH 5.5 and 20% PEG 3000 (Figure S1). Crystals were not cryo-protected as PEG 3000 presence prevented ice formation. Crystal images and dimension measurements were taken using a Rock Imager 1000 automated imaging system (Formulatrix). X-ray diffraction data of *Bp*BOD were collected at beamline P14, operated by EMBL Hamburg at the PETRA III storage ring (DESY, Hamburg, Germany) using a Dectris Eiger-16 M detector at 0.97625 Å wavelength. Diffraction data were indexed, integrated, and reduced with XDS [36] and Scala [37]. The structure was solved by molecular replacement using Phaser and the coordinates of *Bp*BOD model were based on CotA laccase from *Bacillus subtilis* (PDB 4Q8B). Refinement was performed using PHENIX [38], and manual model building, real-space refinement, and structure validations were performed using Coot [39]. Graphical presentations were generated using Pymol [40]. Crystal parameters and data statistics are summarized in Table 1. The *Bp*BOD dimeric interphase was calculated and analyzed using PDBePISA [41]. Evolutionary conservation estimation (Figure S2) was performed using the ConSurf server [42].

### 2.4. Bilirubin Oxidase Structure Analysis

*Bp*BOD crystal parameters and data statistics are summarized in Table 1. Evolutionary conservation estimation in Figure S2 was performed using ConSurf server [42]. Graphical representations of *Bp*BOD disulfide bond location, cysteines distribution, and surface charge distribution (Figures S3–S5) were prepared using PyMol [40].

### 2.5. Bilirubin Oxidase-Based Cathode Fabrication

Clean GCEs were washed with 70% ethanol followed by washing with water and then dried under atmospheric conditions. Five microliters of MWCNT suspension was deposited on the GCEs, which were then dried in a vacuum at room temperature for 30 min. The GCE/MWCNTs were then modified with the *Bp*BOD enzyme. The stock solutions of TCEP and PyMal were prepared (200 mM and 1.4 mM, respectively, dissolved in DMF). A mixed solution containing the enzyme, TCEP, and PyMal was prepared by mixing the enzyme and the TCEP and incubating for 20 min before the PyMal addition. The BOD/TCEP-PyMal mixture was incubated for more than 2 h before the deposition on the electrode. The final concentrations of the *Bp*BOD, TCEP, and PyMal in the solution were 28 µM, 10 mM, and 67 µM, respectively. Five microliters of the solution was then deposited on the MWCNT-modified GCEs followed by drying in air for 1 h. An additional layered configuration was examined. For the electrode preparation, the PyMal stock solution was diluted 20-fold and 5 µL of the solution was deposited on the GCE/MWCNTs. The electrode was then dried under air. In the next step, 5 µL of the reduced enzyme mixed solution was deposited on the electrode. Cyclic voltammetry measurements were performed to evaluate the enzyme’s electrochemical performance. Measurements were performed in PB 0.1 M pH 7.4 at 45 °C under an oxygen saturated atmosphere, using a scan rate of 10 mV/s. To verify that PyMal is indeed linked to the *Bp*BOD enzyme, a mixed solution was prepared in the same way, except 0.5 µL of 2-mercaptoethanol was added to the reduced *Bp*BOD enzyme. Five microliters of the mixed solution was then deposited on the GCE/MWCNTs and the electrode was dried under air. Cyclic voltammetry measurement results are presented in Figure S6.

### 2.6. Fluorescence Intensity of the BpBOD-Pyrene-Maleimide-TCEP Mixture

The fluorescence intensity of the *Bp*BOD, PyMal, and TCEP mixture during 2 h of incubation was measured in order to estimate the binding of PyMal with the enzyme. The fluorescence of the enzyme/TCEP mixture was measured. As a control, we also followed the sole PyMal solution (in the same concentration). The results are presented in Figure S7. The fluorescence intensities of the *Bp*BOD and PyMal lacking the TCEP were measured as well; the results are presented in Figure S8.

### 2.7. Estimation of the BpBOD Deposited in Electrochemically Active Orientation on the Electrode

For the estimation of the amount of the protein deposited on the electrode, an aqueous solution of ABTS (100 µM) was prepared. A 96-well plate was used for calibration curve preparation. First, 200 µL of the ABTS solution was placed in each test well. Then, different amounts of the *Bp*BOD solution were added to each test well containing the ABTS solution. The *Bp*BOD quantities added to each test well varied from 0 to 0.005 mg. After 2 min of incubation, the green color obtained from the ABTS reduction by the *Bp*BOD was quantified by the absorbance read at 420 nm [12,43,44]. The calibration curve is presented in Figure S9. To estimate the amount of enzyme oriented in electrochemically active form, GCE/MWCNTs/*Bp*BOD, GCE/MWCNTs/TCEP-*Bp*BOD, GCE/MWCNTs/TCEP-PyMal-*Bp*BOD as a mix, GCE/MWCNTs/TCEP-PyMal-*Bp*BOD as layers, GCE/MWCNTs/PyMal-*Bp*BOD without TCEP as a mix and GCE/MWCNTs/PyMal-*Bp*BOD without TCEP as layers modified electrodes were incubated in PB, pH 7.4 for one minute; then, 100 µM of ABTS solution was added and the electrodes were incubated for 2 min; the green color obtained was quantified at 420 nm. The amount of the protein released into the ABTS solution was estimated according to the calibration curve. Then, the *Bp*BOD amount deposited on the electrode surface was estimated by subtracting the protein amount released off the electrode from the protein amount that was deposited on the electrode during its preparation. The results are summarized in Table S1.

### 2.8. Electron Transfer Rate (Ket) Calculations

The estimation of the electron transfer rate was performed according to the following formula [45]:

(1) $$Ket=\frac{j_{max}}{z\times F\times \Gamma_{Bp\mathrm{BOD}}}$$

where *j_max_* is the maximal current density obtained in the cyclic voltammetry measurements, *z* is the number of electrons involved in the reduction reaction, *F* is the Faraday constant and *Γ_Bp_*_BOD_ is the electrode surface area covered by each mole of the enzyme, according to the data presented in Table S1. The *Ket* values obtained are summarized in Table S2.

### 2.9. Effect of TCEP and PyMal on BpBOD Activity

To estimate the effect of TCEP and PyMal on *Bp*BOD activity, mixtures lacking or consisting of the enzyme, the TCEP, or the PyMal were prepared. The *Bp*BOD concentration in all the mixtures was 28 µM. For the *Bp*BOD/TCEP/PyMal preparation, the enzyme was incubated with the TCEP for 20 min before the PyMal addition. Then, the mixture was incubated for an additional two hours. For the estimation of the enzyme activity, 2 µL of each mixture was added to 198 µL of 100 µM ABTS solution in a 96-well plate, and the absorbance at 420 mn was then measured every 10 s for 10 min. The results are presented in Figure S10.

## 3. Results and Discussion

### 3.1. BpBOD Structure Revealed a Unique Disulfide Bond

**Figure 2:**
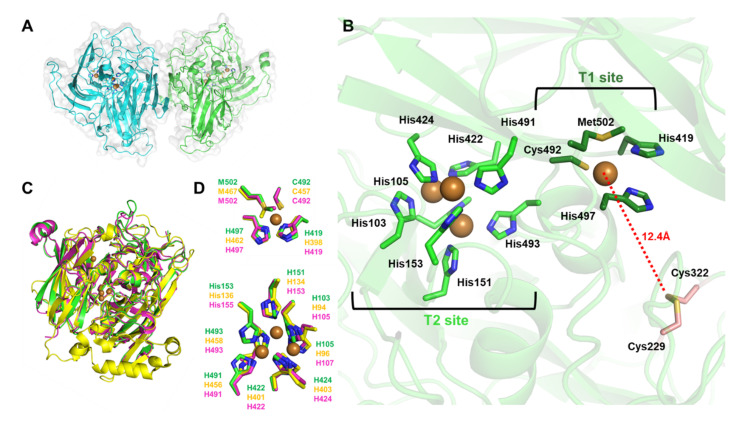
Crystal structure of *Bp*BOD (**A**) The overall structure of *Bp*BOD (PDB: 7Z5P) is presented in green and cyan cartoons with grey surface representation, indicating the two monomers forming the homodimer. Copper ions are presented as brown spheres and the chelating residues are presented in green and cyan sticks. (**B**) Multicopper sites and *Bp*BOD unique disulfide bond (Cys229-Cys322 in pink sticks). T1 and T2/T3 site chelating residues are presented in dark green and light green sticks, respectively. The distance between the T1 copper ion and the disulfide bond is marked by the red dash line. (**C**) Multiple structural alignments of *Bp*BOD and similar enzymes. *Bp*BOD is presented in green, BOD from *Myrothecium verrucaria* (*Mv*BOD) is presented in yellow, and CotA laccase from *Bacillus subtilis* (*Bs*CotA) is presented in magenta. The structures share RMDS in the range of 0.336–0.811. (**D**) Structural alignment of T1 (up) and T2/T3 (down) sites copper chelating residues of *Bp*BOD (green), *Mv*BOD (yellow), and *Bs*CotA (magenta). The residue numbering is colored accordingly.

To determine the *Bp*BOD structure, the recombinant enzyme, comprising 509 amino acids, was overexpressed in *E. coli* BL21 (DE3) cells under micro-aerobic conditions enriched with CuSO_4_ [12,17]. *Bp*BOD was extracted from soluble bacterial lysate and purified in sufficient amounts (7.5 mg pure *Bp*BOD per 1 L cell culture) using affinity and size-exclusion chromatography (see experimental section and Supplementary Information, Figure S1). Pure fractions of active *Bp*BOD, as evidenced by a turquoise color upon addition of ABTS (Figure S1), were used for crystallization screening, with the first crystals appearing after several months at 20 °C. The crystallization conditions were optimized and optimized crystals were used for X-ray data collection. The structure of *Bp*BOD was solved at 3.5 Å resolution presenting a homodimer, with 20,637 Å^2^ interface (PDB 7Z5P, Figure 2A). Crystal parameters and data statistics are summarized in Table 1. Interestingly, the *Bp*BOD core was found to be more structured compared to the enzyme peripheral regions. As depicted, long and unstructured loops “cover” the catalytic sites, representing a large portion of the enzyme surface. Each monomer contains multi-copper T2/T3 and T1 sites having three and one copper ions, respectively. T2/T3 site residues His103, His105, His151, His153, His422, His424, His491, and His493 chelate three Cu^2+^ ions and exhibit high evolutionary conservation (Figure S2). The adjacent T1 site is comprised of residues His419, Cys492, His497, and Met502 that coordinate a single Cu^2+^ ion at the enzyme C’-terminal region. The revealed oxidation state of 4 Cu^2+^ can be correlated with the enzyme resting state [8]. The cysteine pair Cys229 and Cys322 was found to form a disulfide bond, located 12.4 Å away from the T1 site copper ion (Figure 2B, and Figure S3). In total, the *Bp*BOD sequence comprises four cysteines: one disulfide bond pair, one T1 site chelating residue (Cys492), and one oxidized Cys146 located 12 Å from the nearest T2/T3 site Cu^2+^ ion (Figure S4).

**Table 1:** X-ray data collection and refinement statistics.

Protein [PDB Code]	*Bp*BOD [7Z5P]
**Data collection**	
**Synchrotron**	DESY, Hamburg
**Beamline**	P14
**Wavelength**	0.97625
**Space group**	C 1 2 1
Resolution range *	59.22–2.991 (3.098–2.991)
**Unit cell dimensions a, b, c (Å)** **α, β, γ (°)**	198.96, 63.51, 115.96 90, 124.567, 90
Total reflections *	166,046 (10,488)
Unique reflections *	24,048 (2229)
Completeness (%) *	98.12 (92.57)
Mean I/sigma(I) *	7.4 (1.5)
**Wilson B-factor**	78.94
R-merge *,†	0.188 (1.47)
R-meas *	0.204 (1.60)
CC_1/2_ *	99.5 (71.9)
**Refinement**	
**Resolution (Å)**	59.22–3.5
Reflections used in refinement *	23,986 (2219)
Reflections used for R-free *	2398 (221)
***R/R*** **_free_** ‡,*	0.254/0.267 (0.491/0.479)
**Number of non-hydrogen atoms**	8244
**Macromolecules**	8236
**Ligands**	8
**Protein residues**	1010
**RMSD bonds length (Å)**	0.009
**RMSD bond angles (** **°** **)**	1.45
**Ramachandran favored (%)**	90.18
**Ramachandran allowed (%)**	8.62
**Ramachandran outliers (%)**	1.20
**Average B-factor (Å^2^)**	90.64
**Macromolecules**	90.63
**Ligands**	101.28

* Values in parentheses correspond to the highest resolution shell. † *R*_merge_ = ∑_hkl_∑*_i_*|*I_i_*(hkl) − *I*(hkl)〉|/∑_hkl_∑*_i_I_i_*(hkl), where *I* is the observed intensity, and <*I*> is the mean value of *I*. ‡ *R/R*_free_ = ∑_hkl_||F_obs_| − |F_calc_||/∑_hkl_|F_obs_| where *R* and *R*_free_ are calculated using the test reflections, respectively. The test reflections (5%) were held aside and not used during the entire refinement process.

**Figure 3:**
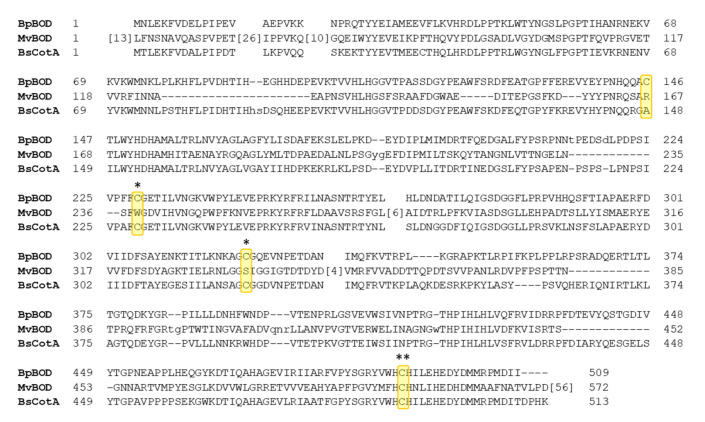
Multiple sequence alignment of *Bp*BOD related enzymes. *Mv*BOD represents BOD from *Myrothecium verrucaria* and *Bs*CotA represents CotA laccase from *Bacillus subtilis*. The cysteine residues in the *Bp*BOD are presented in yellow, including disulfide bond participating residues (*) and the T2/T3 copper chelating residue (**). The numbers in brackets represent the number of residues with no sequence homology among the sequences that were excluded from the graphical comparison (generated using Clustal Omega online tool) [47].

Structural alignment of *Bp*BOD with BOD from *Myrothecium verrucaria* (*Mv*BOD, PDB 2XLL) and CotA laccase from *Bacillus subtilis* (*Bs*CotA, PDB 4Q8B) showed a moderate overall resemblance between the enzyme folds, with high similarity around the T2/T3 site (Figure 2C,D) [33,46]. *Mv*BOD possesses an extended C’-terminal domain when compared to the *Bp*BOD sequence (Figure 3). Moreover, the multiple sequence alignment emphasized the exclusive disulfide bond in *Bp*BOD, which is not found in *Mv*BOD (Figure 3). A surface charge distribution map of *Bp*BOD revealed that the disulfide bond is found in a negatively charged region at the enzyme surface (Figure S5).

### 3.2. BpBOD Site-Specific Direct Electron Transfer

To date, none of the solved BOD structures have originated from a bacterial source [48]. While the solved *Bp*BOD structure has high homology to previously solved structures, it has a unique surface-exposed disulfide bond in the vicinity of the T1 site (Figure 2 and Figure 3). These cysteine residues may be exploited for site-specific immobilization of *Bp*BOD with the electrode surface. This may lead to improved ordered enzyme orientation towards the electrode with a short electron transfer distance from the BOD T1 site. Recently, BOD from *Magnaporthe oryzae* (*Mo*BOD) was rationally designed to display a mutated cysteine residue using site-directed mutagenesis. The cysteine-maleimide reaction allowed a fast and efficient methodology to bind and orient the *Mo*BOD T1 site toward the electrode surface [49]. The crystal structure of the *Bp*BOD revealed a native disulfide bond close to the T1 site and therefore can serve as a promising candidate for improved immobilization with electrodes. Since the *Bp*BOD disulfide bond is located between two unstructured loops, we assumed that any electrode-enzyme interactions would be easily established with minimal interference due to the region’s higher flexibility.

In previously published research, the electron transfer process between the BOD and electrodes was improved by using hydrophobic conjugated chemicals or hydrogels [9,25,50,51,52,53]. While hydrophobic interactions can optimize the BOD orientation and its electron transfer rate, a covalent bond formation has further advantages in terms of long-term stability and irreversible orientation over adsorption.

**Figure 4:**
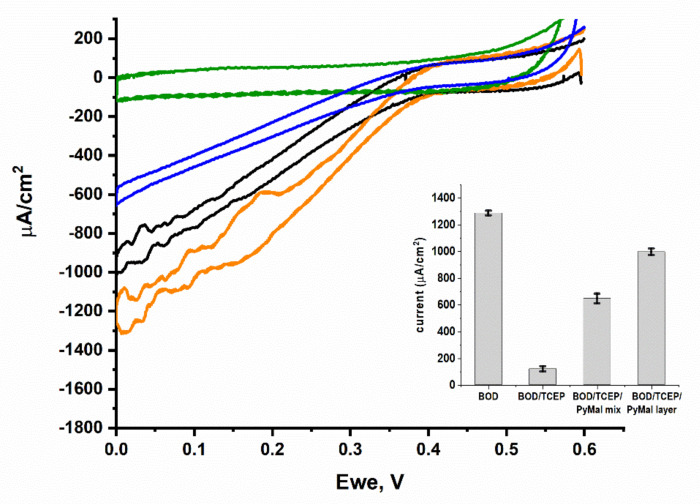
Bioelectrocatalytic activity of *Bp*BOD immobilized on GCE by various methods. Cyclic voltammogram of: GCE/MWCNTs/*Bp*BOD (orange), GCE/MWCNTs/*Bp*BOD, TCEP (green), GCE/MWCNTs/*Bp*BOD, TCEP, PyMal as a mix (blue), GCE/MWCNTs/*Bp*BOD, TCEP, PyMal as layers (black). Measurements were performed in PB 0.1 M pH 7.4, at 45 °C, under a saturated oxygen atmosphere. A scan rate of 10 mV/s was used. Error bars represent the standard error from three independently prepared samples at 0 V vs. Ag/AgCl.

**Figure 5:**
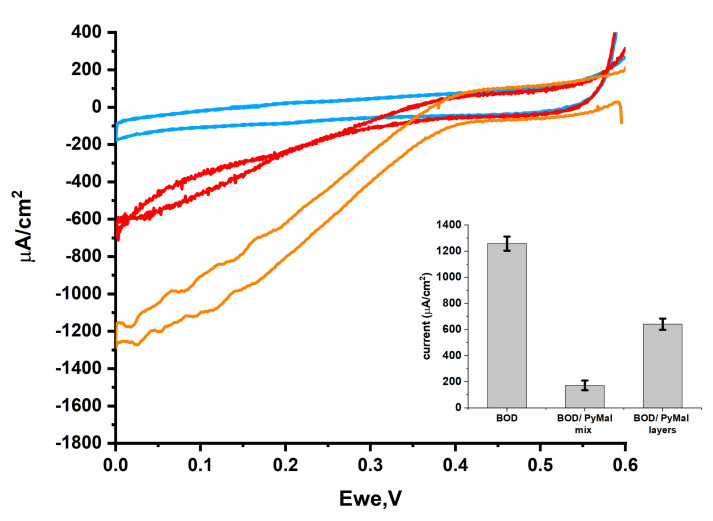
Bioelectrocatalytic activity of *Bp*BOD immobilized on GCE without TCEP. Cyclic voltammetry measurement curve of BOD deposited on GCE/MWCNTs (orange), GCE/MWCNTs modified with a solution containing PyMal and BOD as a mix (blue) and as layers (red). Measurements were performed in PB 0.1 M pH 7.4 at 45 °C, under an oxygen saturated atmosphere, using a scan rate of 10 mV/s. Error bars represent the standard error from three independently prepared samples at 0 V vs. Ag/AgCl.

To study the site-specific immobilization of *Bp*BOD, the disulfide bond between Cys229 and Cys322 was chemically reduced using a TCEP reagent. Unlike the commonly used dithiothreitol (DTT), TCEP cannot further react with available maleimide moieties and therefore was preferred for this task. The TCEP-reduced *Bp*BOD enzyme was then linked to PyMal using a Michael’s addition reaction [49]. The mixture was deposited on a GCE modified with MWCNTs. This configuration was annotated as a mixed configuration. Alternatively, we examined a layered configuration that was constructed by depositing the TCEP-*Bp*BOD mixture on a PyMal modified GCE/MWCNTs: this configuration was annotated as a layered configuration. To explore how the different configurations affect the biocathode performance, we followed the generated bioelectrocatalytic currents under oxygen saturated conditions at 45 °C (Figure 4). Using the layered and mixed configurations, the tested electrodes developed bioelectrocatalytic currents of 1000 µA/cm^2^ and 700 µA/cm^2^ with an onset potential at 0.4 V vs. Ag/AgCl, respectively. By excluding the PyMal molecule, significantly lower bioelectrocatalytic currents of ~100 µA/cm^2^ were obtained (Figure 4, green curve). These results suggest that the PyMal contributes to the *Bp*BOD proper orientation toward the electrode surface and improves the biocathode performance. By excluding the TCEP reduction step, the PyMal/*Bp*BOD layered configuration has led to a ~50% decrease in the generated bioelectrocatalytic currents (Figure 4 and Figure 5). A similar trend was observed with the mixed configuration. As depicted in Figure 4, a ~four-fold decrease of the bioelectrocatalytic currents was observed while only TCEP was added to the *Bp*BOD without the addition of the PyMal. These results support the hypothesis that the free cysteines contribute to proper *Bp*BOD immobilization. Moreover, the TCEP treatment dramatically affects the enzyme bioelectrocatalytic activity (Figure 5). The obtained results suggest that the improved *Bp*BOD bioelectrocatalytic currents are due to the PyMal-specific binding, which occurs through the reduced disulfide bond and electrostatic interactions. To elucidate if the maleimide moiety indeed contributes to the enzyme binding or rather it derives from electrostatic interactions, we designed an additional configuration where the maleimide group is chemically blocked. This was achieved by reacting the maleimide moiety with mercaptoethanol prior to the enzyme addition. The obtained results exhibit significantly lower bioelectrocatalytic currents (Figure S6). To further understand the nature of the interactions between the *Bp*BOD and the PyMal, we used fluorescence measurements. Pyrene is a highly fluorescent molecule that can be easily followed spectroscopically. Thus, we examined the close interaction between the T1 copper center (with the disulfide bond) and the maleimide moiety by following the fluorescence energy transfer quenching. For that, we measured the decay of the fluorescence emission in the presence and the absence of the TCEP. As depicted in Figure S7, we observed decay in the fluorescence signal in the presence of the TCEP. A similar configuration lacking the TCEP reduction step did not depict any decay over time (Figure S8). These results imply that the reduced disulfide bond indeed enables close proximity to the pyrene maleimide moiety, which results in a quenching effect.

To further characterize the *Bp*BOD-based systems, we determined the enzyme loading on the electrode surface. Following enzyme immobilization, the bonded enzyme (that remained on the electrode surface) was immersed in an ABTS solution. The results were compared to dissolved free *Bp*BOD protein using a calibration curve (assuming similar activity, see Supplementary Materials). These results allowed us to further calculate the *Ket* using the bioelectrocatalytic current saturation and enzyme loading (Table S2). As shown, in the absence of the TCEP, the *Ket* of both the layered and mixed configurations dropped by ~1.5-fold. The drop was intensified while the bioelectrocatalytic currents were measured, and ~1.6 and 3-fold decreases of the bioelectrocatalytic currents were observed in the layered and mixed configuration, respectively. Untreated *Bp*BOD deposited on the MWCNTs electrode exhibited higher bioelectrocatalytic currents as compared with the treated one (Figure 4, Table S2). By examining the obtained results, we can conclude that the TCEP reduction process contributes to the maleimide conjugation with *Bp*BOD, which in turn improves the biocathode performance. On the other hand, the TCEP addition has a negative effect on the bioelectrocatalytic activity. As shown before, disulfide bonds play an important role in enzymatic structural stability [54,55]. The TCEP reduction process at the *Bp*BOD disulfide position might cause a structural rearrangement in the enzyme folding, which in turn affects its catalytic performance. Furthermore, structural rearrangement may affect the electron transfer distance between the T1 site and the electrode, or between the T1 and the T2/T3 sites, which in turn affects the internal electron transfer process rate.

As shown in Figure 2A, the *Bp*BOD crystal structure reveals a dimeric form. The homodimeric structure may hinder the proper orientation of both subunits on the electrode surface while attached to the PyMal. This leads to at least two different BOD populations, one properly oriented with a short distance to the electrode, and the other with slow and inefficient electron transfer. By examining the results summarized in Table S2, we can observe that while the bioelectrocatalytic current achieved with the mixed or the layered configurations is higher (in the presence of the TCEP), the enzyme loading is relatively high as well, which resulted in less dominant *Ket* values that reached 8 s^−1^ in the layered configuration. Furthermore, our results imply that the PyMal improves the electron transfer process to the T1 site, as can be seen in Figure 4. We hypothesize that while part of the *Bp*BOD enzymes establish short electron transfer distances from the T1 site, which in turn reach high *Ket*, the far end subunits are wrongly orientated and do not contribute to the bioelectrocatalytic process. These wrongly oriented subunits are still active, and can therefore oxidize the ABTS and raise the measured enzyme content on the electrodes. The outcome leads to a lower average *Ket*. Taking into account the different parameters described above, we can conclude that while site-specific integration has advantages, it also leads to drawbacks in terms of *Ket*. Previously published work that presented site-specific immobilization of BOD on electrode surfaces revealed a similar trend of lower bioelecterocatalytic currents as compared with standard BOD/MWCNTs configurations [49]. Further spectroscopic measurements, electron paramagnetic resonance (EPR), or transient absorbance measurements may be applied to fully elucidate this phenomenon and enable improved efficiency.

## 4. Conclusions

We determined the first crystal structure of bilirubin oxidase from a bacterial source, isolated from the thermophilic *Bacillus pumilus*. The *Bp*BOD dimeric structure exhibits a solvent-exposed disulfide bond formed between Cys229 and Cys322 and positioned in the vicinity of the enzyme’s T1 site. We investigated different approaches to incorporate *Bp*BOD with electrodes, exploiting the disulfide bond for site-specific immobilization. *Bp*BOD disulfide bond reduction with TCEP in conjugation with a deposited PyMal electrode enhanced the bioelectrocatalytic currents by 50%. Furthermore, we showed that the TCEP reduction step improves binding to the maleimide moiety, which in turn enhances the biocathode performance. On the other hand, reducing the disulfide bond suppresses the *Bp*BOD activity on the electrode surface for both the layered and the mixed configurations and the *Bp*BOD homodimeric form can lead to wrongly oriented subunits that do not contribute to bioelectrocatalysis. The presented work opens a promising route for the rational design of *Bp*BOD configurations to allow enhanced DET capabilities and oxygen reduction efficiency.

## Data Availability

Data available on request from the authors.

## Supplementary Materials

The following supporting information can be downloaded at: https://www.mdpi.com/article/10.3390/bios12050258/s1. Figure S1, The expression, purification, and crystallization of *Bp*BOD. (A) A chromatogram depicting Ni-NTA affinity purification of *Bp*BOD. (M) protein marker in kDa. FT represents flow-through fractions; Wash represents washing fractions after loading. Elution fractions are presented according to their imidazole concentration during elution (60, 260, and 500 mM). Elution fractions using 260 mM imidazole were combined, concentrated, and used for sizeexclusion chromatography. (B) Size exclusion purification of *Bp*BOD. (M) protein marker in kDa. The eluted fractions are marked in red on the chromatograph and their corresponding SDS-PAGE analysis is presented to the right. (C) Cell extract-based *Bp*BOD activity test. Soluble lysates from *E. coli*/pET21a (left) and *E. coli*/pET21a-*Bp*BOD (right) were supplemented with ABTS solution at RT. Turquoise pigmentation confirms the *Bp*BOD-containing sample. (D) *Bp*BOD activity test for purification fractions. Elution fractions from the Ni-NTA column were incubated with ABTS solution at RT. *Bp*BOD crystals representations within their crystallization drops are shown in panels (E) and (F), while a “close-up” capture of typical *Bp*BOD is presented in panel G). *Bp*BOD crystal average diameter was measured and presented in a red line and white box, Figure S2. ConSurf server output for the conservation score of *Bp*BOD. ConSurf detected 510 sequence model hits. The calculation is performed on a sample of 150 sequences that represent the list of unique homologues to the query, Figure S3. The disulfide bond location in *Bp*BOD structure. (A) The overall location of *Bp*BOD disulfide bond. One oxidized disulfide bond was found per monomer. The bond is located 12.4 Å away from the T1 copper ion. The 2mFo-DFc electron density map contoured at 1 σ around the disulfide bond in chain A (B) and chain B (C) is presented as blue mesh, Figure S4. The cysteines distribution in *Bp*BOD structure. *Bp*BOD sequence comprised of 510 amino acids including 4 cysteines; Cys492 which chelates the copper ion in T1, Cys229, and Cys322 which participate in the disulfide bond, and Cys146. Compared with the disulfide bond, Cys146 is located in a more buried region with reduced solvent accessibility, Figure S5. Surface charge distribution in *Bp*BOD structure (red positively charged, blue negatively charged). The copper ions are presented as brown spheres. The disulfide bond is presented in sticks, Figure S6. Cyclic voltammetry measurement curves of MWCNTs/GCE modified with a mixed solution prepared with 2-mercaptoethanol. Measurement was performed in PB 0.1 M pH 7.4 at 45 °C, under an oxygen saturated atmosphere, using a scan rate of 10mV/s. Error bars represent the standard error from three independently prepared samples at 0V vs. Ag/AgCl, Figure S7. Fluorescence of *Bp*BOD, TCEP, and PyMal. Measurements were performed during two hours of incubation. *Bp*BOD/TCEP (yellow), PyMal (black), *Bp*BOD/TCEP/ PyMal after incubation of 30 min (red), 60 min (green), 90 min (blue) and 120 min (orange), Figure S8. Fluorescence of *Bp*BOD and PyMal without TCEP. Measurements were performed during two hours of incubation. *Bp*BOD/ PyMal after incubation of 30 min (blue), 60 min (red), 90 min (black) and 120 min (green), Figure S9. Calibration curve for *Bp*BOD activity using ABTS as a substrate, Figure S10. Estimation of TCEP and PyMal on *Bp*BOD activity. The change in the absorbance at 420 nm during 10 min after the addition of *Bp*BOD (blue), *Bp*BOD/TCEP (orange), *Bp*BOD/TCEP/PyMal (green) and *Bp*BOD/PyMal (red) mixture into ABTS solution, Table S1. Estimation of the *Bp*BOD protein loading on the electrode surface, Table S2. Electron transfer rate (*Ket*) value estimation.
