# Supplementary material for: The Structure of Bilirubin Oxidase from Bacillus pumilus Reveals a Unique Disulfide Bond for Site-Specific Direct Electron Transfer
Source: Biosensors (Basel). 2022 Apr 19;12(5):258. doi: 10.3390/bios12050258 (PMC9138216; doi:10.3390/bios12050258)
Supplement: Supplementary file 1 [file biosensors-12-00258-s001.zip › biosensors-1659128-supplementary.pdf]

biosensors

MDPI

Supplementary

The Structure of Bilirubin Oxidase from *Bacillus pumilus* Reveals a Unique Disulfide Bond for Site-Specific Direct Electron Transfer

Shalev Gihaz [1,†], Nidaa Shrara Herzallh [1,†], Yifat Cohen [1], Oren Bachar [1], Ayelet Fishman [1,*] and Omer Yehezkeli [1,2,3,*]

[1] Department of Biotechnology and Food Engineering, Technion—Israel Institute of Technology, Haifa 3200003, Israel; shalevgihaz@gmail.com (S.G.); nidaa@campus.technion.ac.il (N.S.H.); yifat@bfe.technion.ac.il (Y.C.); orenchus@campus.technion.ac.il (O.B.)

[2] Russell Berrie Nanotechnology Institute, Technion—Israel Institute of Technology, Haifa 3200003, Israel

[3] The Nancy and Stephen Grand Technion Energy Program, Technion—Israel Institute of Technology, Haifa 3200003, Israel

* Correspondence: afishman@technion.ac.il (A.F.); y.omer@technion.ac.il (O.Y.)

† These authors contributed equally to this work.

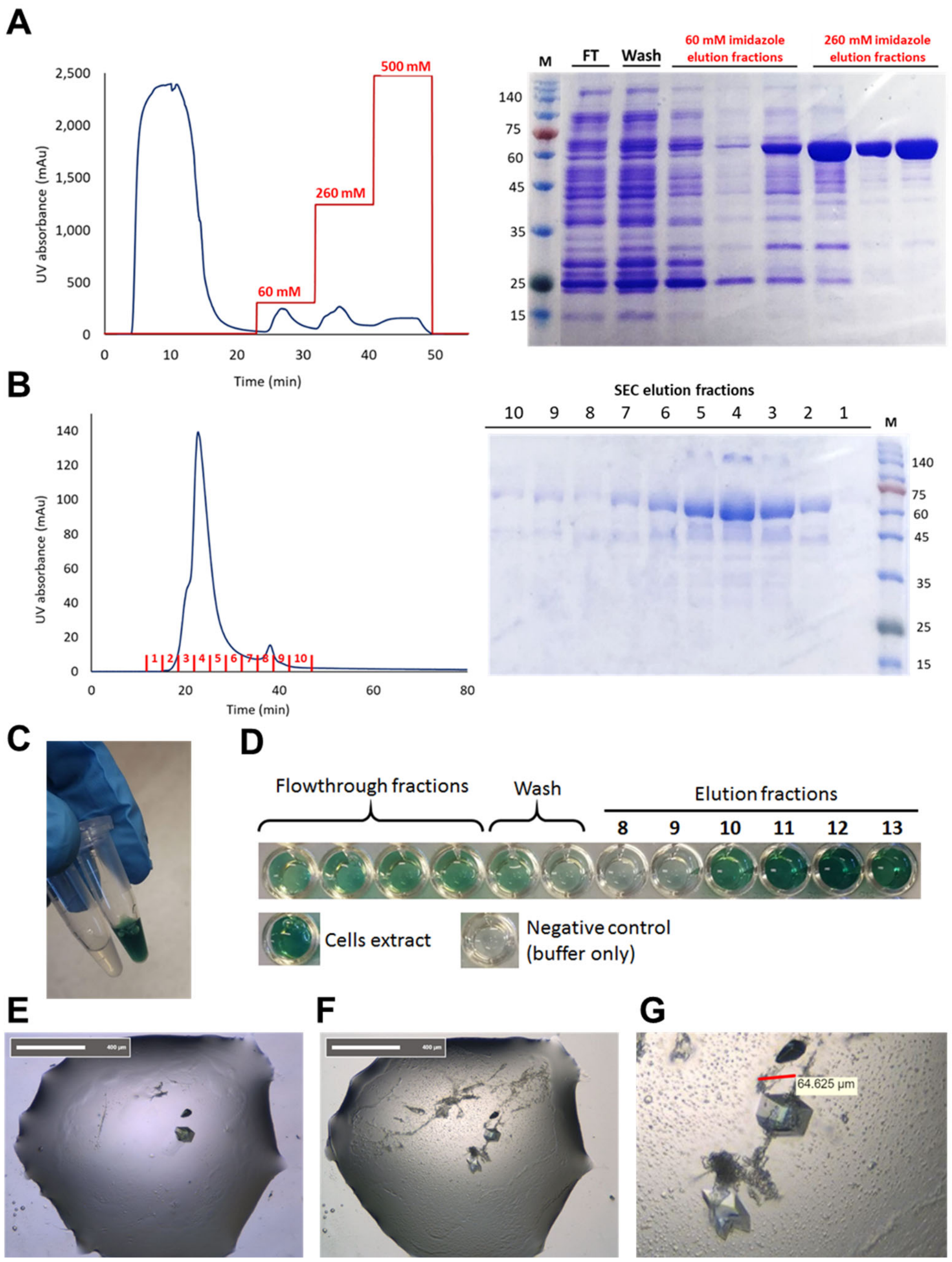

Figure S1. The expression, purification, and crystallization of *Bp*BOD. (A) A chromatogram depicting Ni-NTA affinity purification of *Bp*BOD. (M) protein marker in kDa. FT represents flow-through fractions; Wash represents washing fractions after loading. Elution fractions are presented according to their imidazole concentration during elution (60, 260, and 500 mM). Elution fractions using 260 mM imidazole were combined, concentrated, and used for size-exclusion chromatography. (B) Size exclusion purification of *Bp*BOD. (M) protein marker in kDa. The eluted fractions are marked in red on the chromatograph and their corresponding SDS-PAGE analysis is presented to the right. (C) Cell extract-based *Bp*BOD activity test. Soluble lysates from *E. coli*/pET21a (left) and *E. coli*/pET21a-*Bp*BOD (right) were supplemented with ABTS solution at RT. Turquoise pigmentation confirms the *Bp*BOD-containing sample. (D) *Bp*BOD activity test for purification fractions. Elution fractions from the Ni-NTA column were incubated with ABTS solution at RT. *Bp*BOD crystals representations within their crystallization drops are shown in panels E) and F), while a "close-up" capture of typical *Bp*BOD is presented in panel G). *Bp*BOD crystal average diameter was measured and presented in a red line and white box.

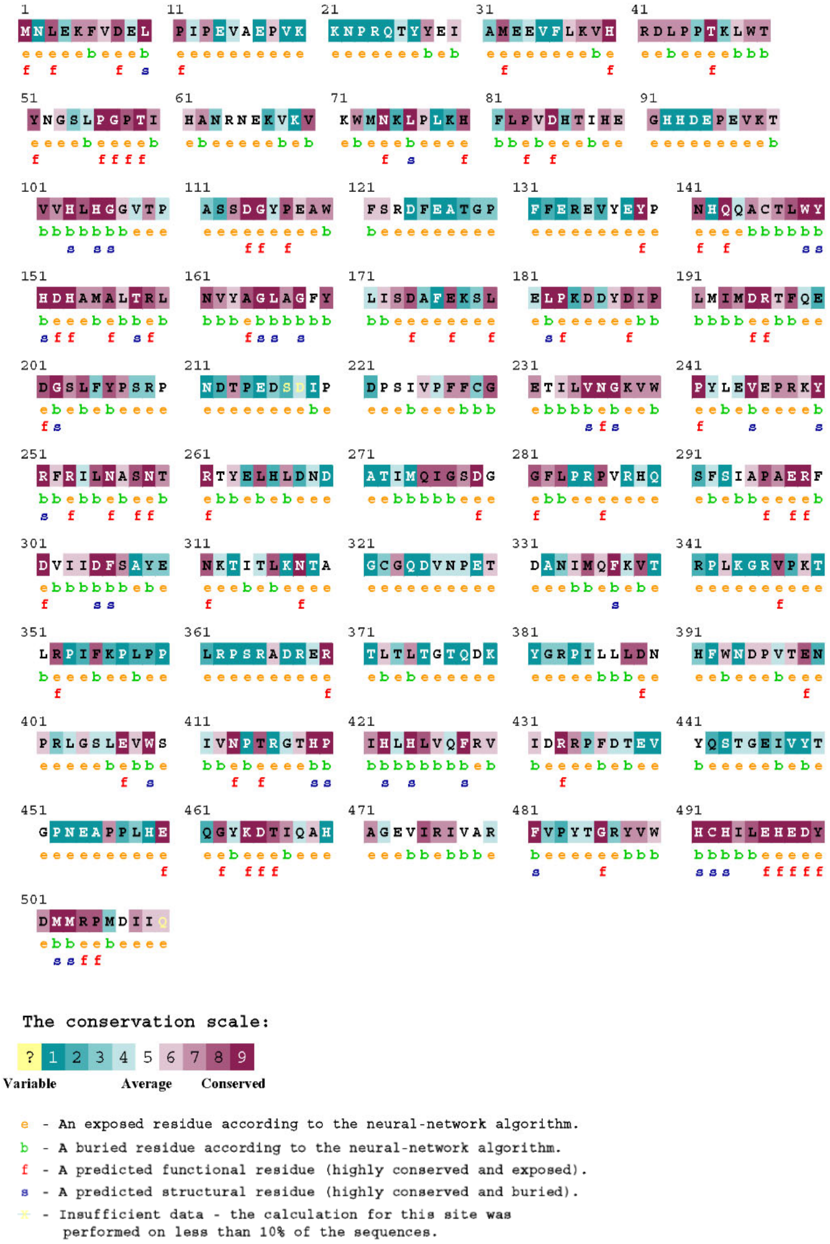

Figure S2. ConSurf server output for the conservation score of *Bp*BOD. ConSurf detected 510 sequence model hits. The calculation is performed on a sample of 150 sequences that represent the list of unique homologues to the query.

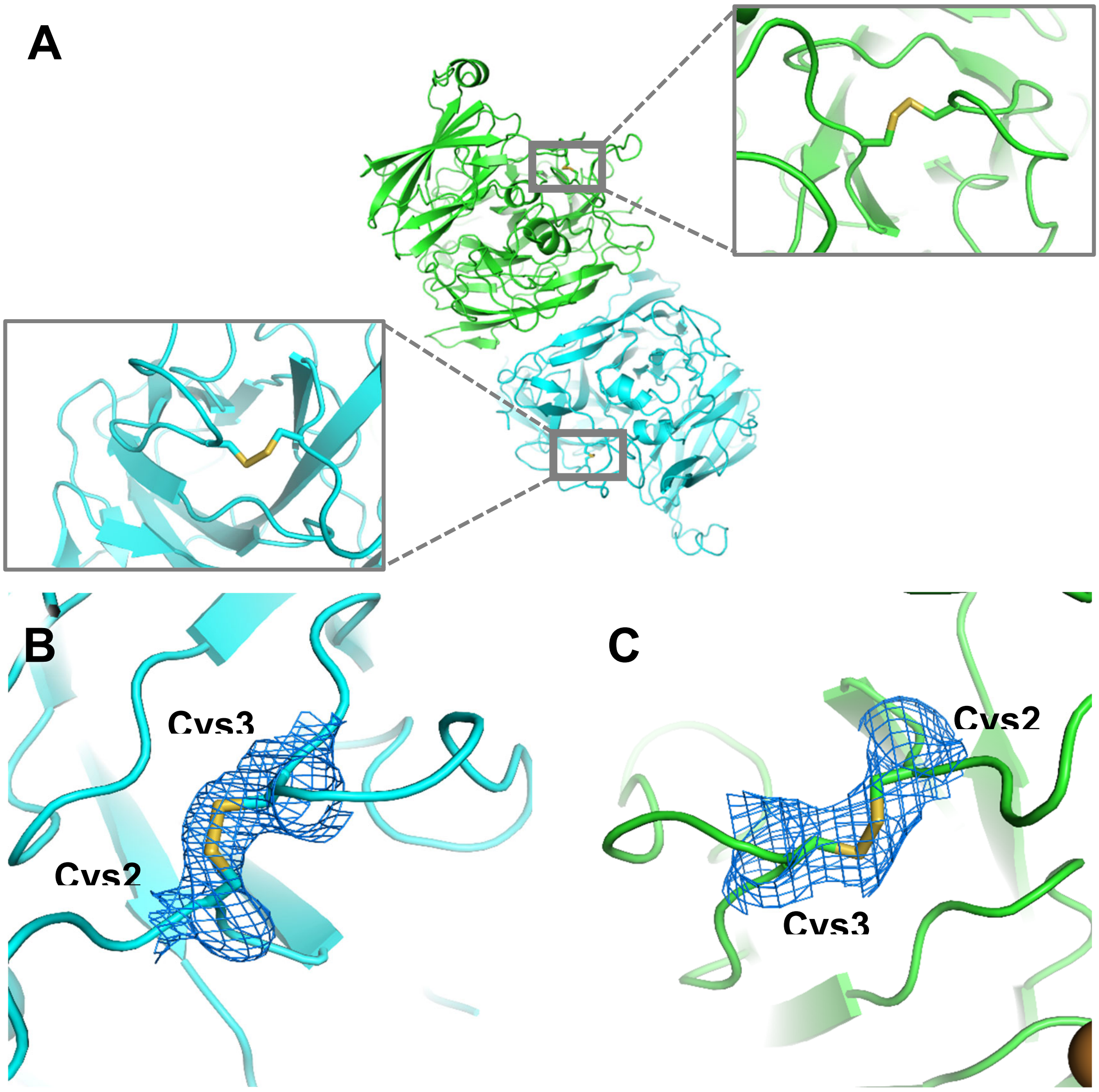

Figure S3. The disulfide bond location in *Bp*BOD structure. A) The overall location of *Bp*BOD disulfide bond. One oxidized disulfide bond was found per monomer. The bond is located 12.4 Å away from the T1 copper ion. The 2mFo-DFc electron density map contoured at 1 σ around the disulfide bond in chain A (B) and chain B (C) is presented as blue mesh.

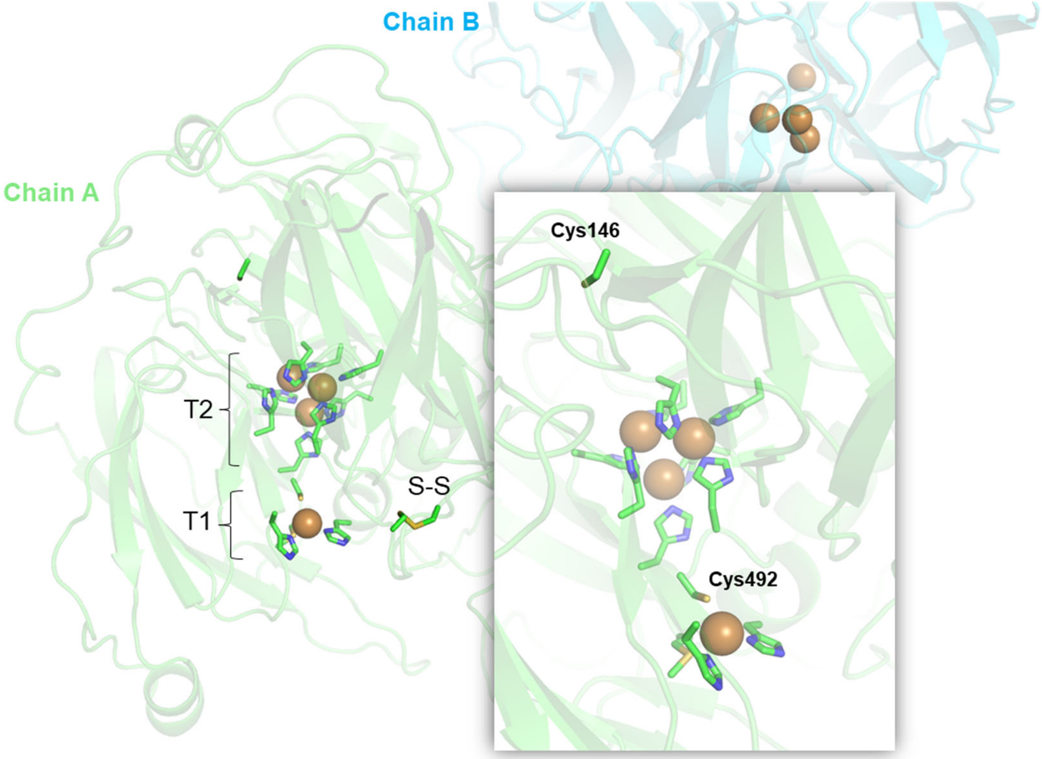

Figure S4. The cysteines distribution in *Bp*BOD structure. *Bp*BOD sequence comprised of 510 amino acids including 4 cysteines; Cys492 which chelates the copper ion in T1, Cys229, and Cys322 which participate in the disulfide bond, and Cys146. Compared with the disulfide bond, Cys146 is located in a more buried region with reduced solvent accessibility.

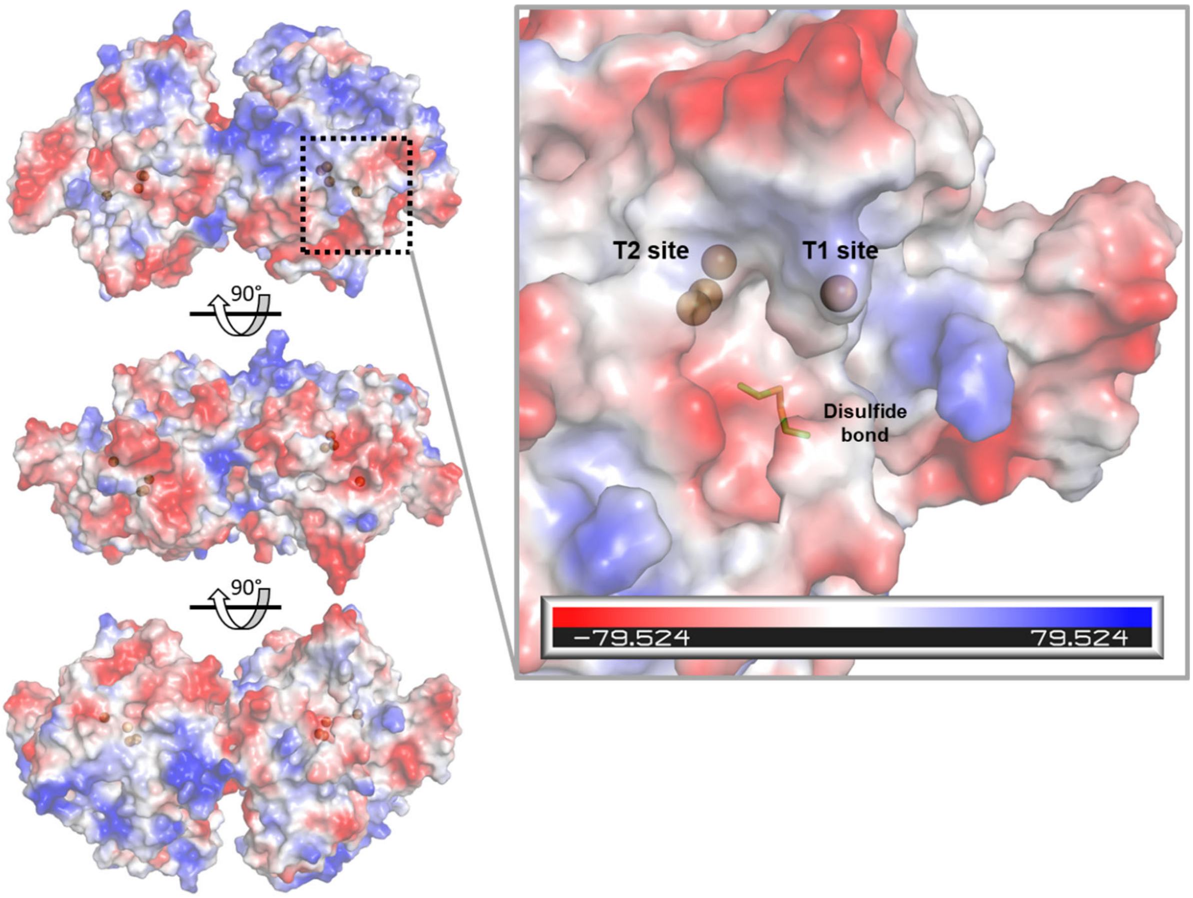

Figure S5. Surface charge distribution in *Bp*BOD structure (red positively charged, blue negatively charged). The copper ions are presented as brown spheres. The disulfide bond is presented in sticks.

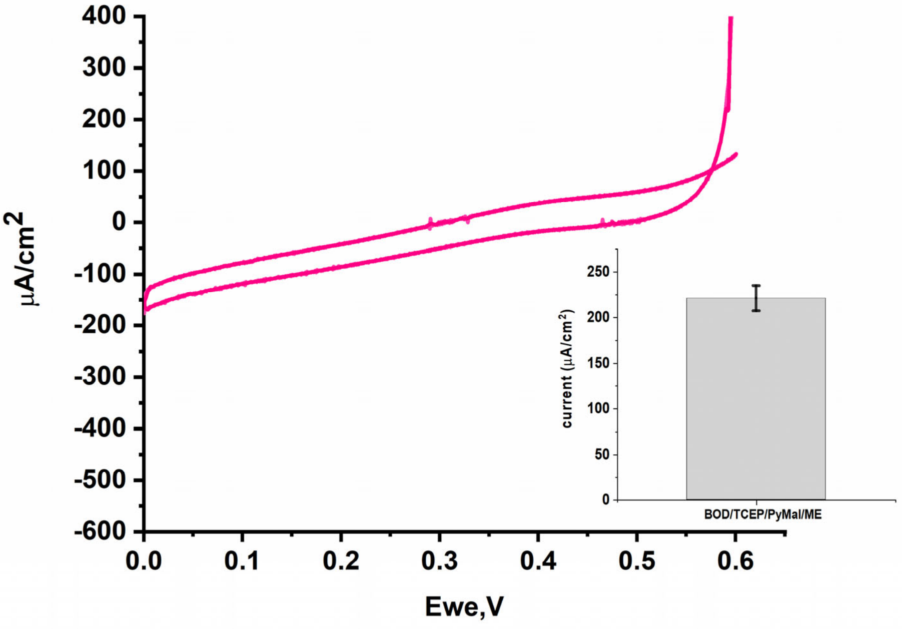

Figure S6. Cyclic voltammetry measurement curves of MWCNTs/GCE modified with a mixed solution prepared with 2-mercaptoethanol. Measurement was performed in PB 0.1 M pH 7.4 at 45°C, under an oxygen saturated atmosphere, using a scan rate of 10mV/s. Error bars represent the standard error from three independently prepared samples at 0V vs. Ag/AgCl.

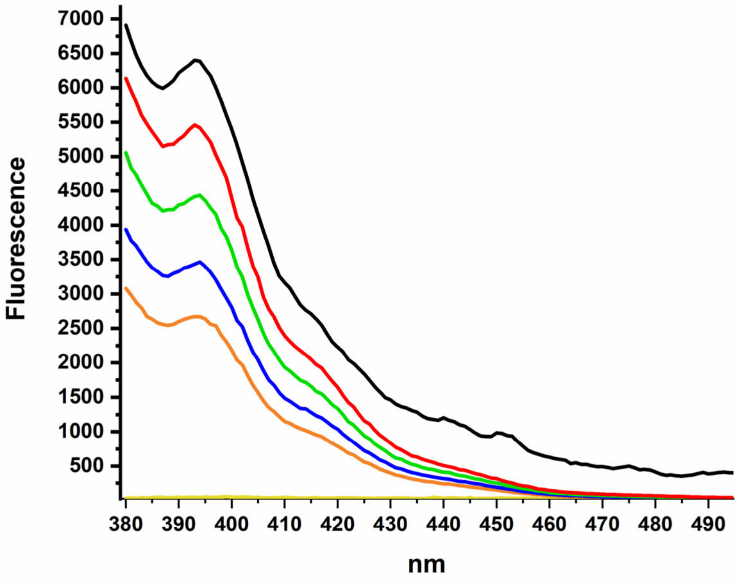

Figure S7. Fluorescence of *Bp*BOD, TCEP, and PyMal. Measurements were performed during two hours of incubation. *Bp*BOD/TCEP (yellow), PyMal (black), *Bp*BOD/TCEP/ PyMal after incubation of 30 min (red), 60 min (green), 90 min (blue) and 120 min (orange).

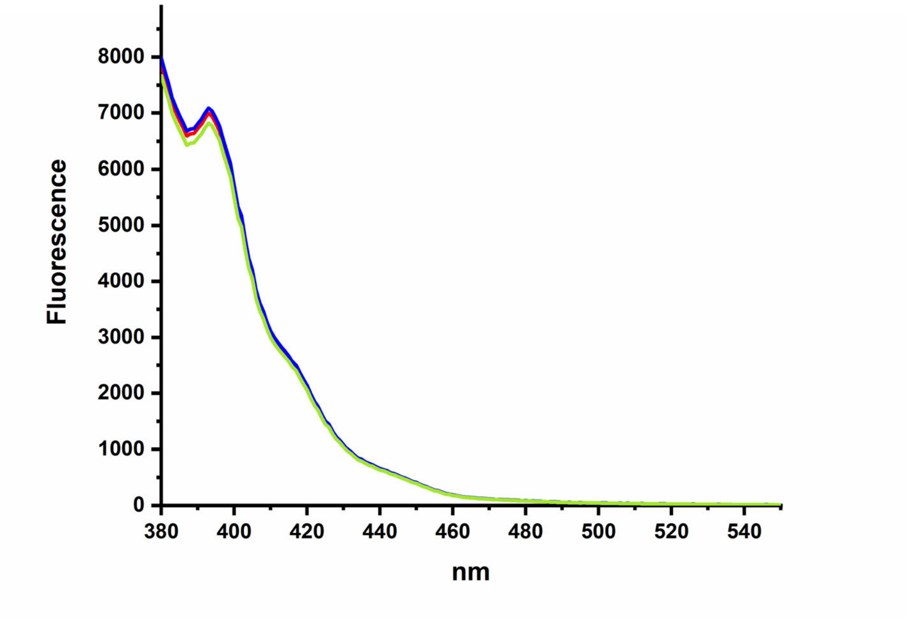

Figure S8. Fluorescence of *Bp*BOD and PyMal without TCEP. Measurements were performed during two hours of incubation. *Bp*BOD/ PyMal after incubation of 30 min (blue), 60 min (red), 90 min (black) and 120 min (green).

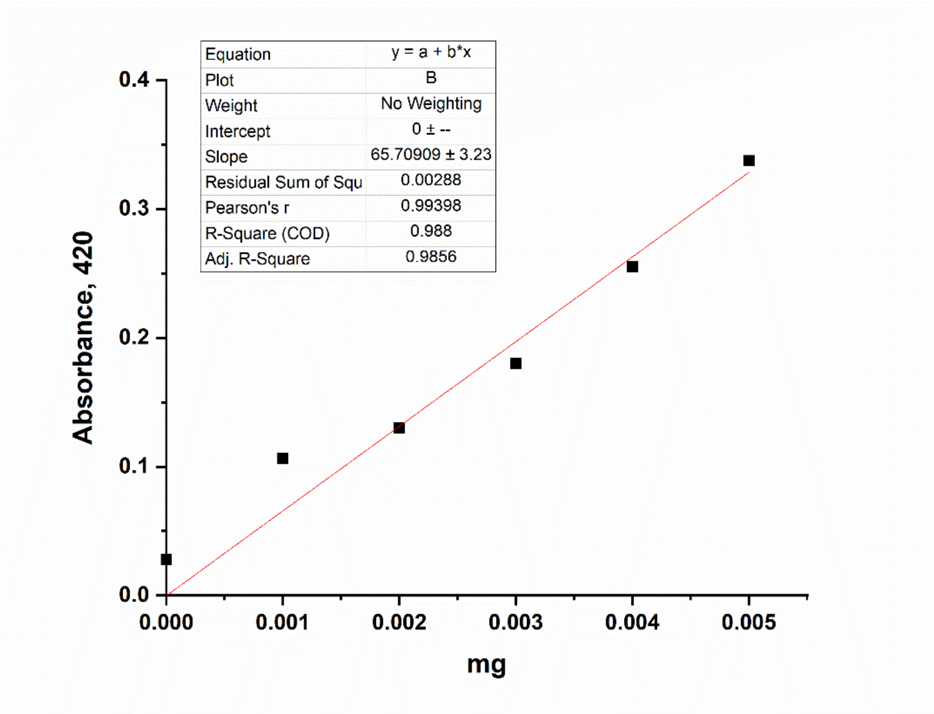

Figure S9. Calibration curve for *Bp*BOD activity using ABTS as a substrate.

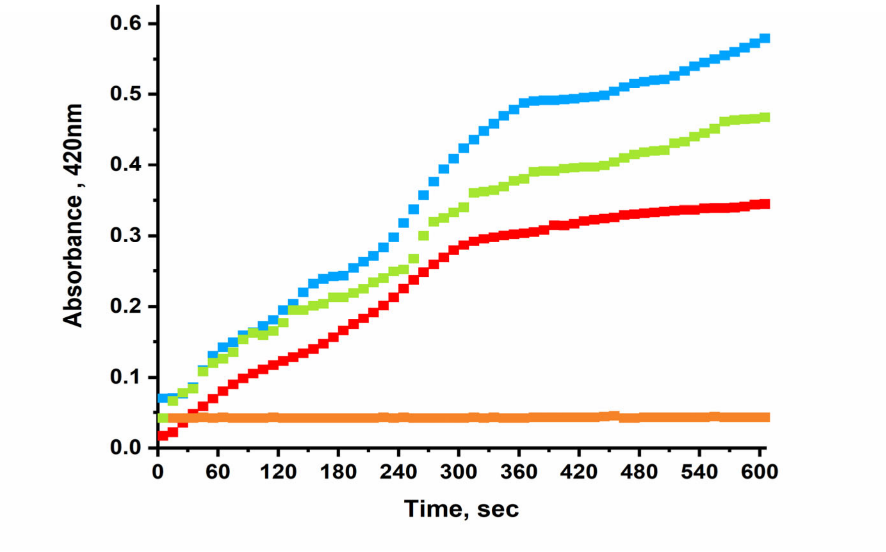

Figure S10. Estimation of TCEP and PyMal on *Bp*BOD activity. The change in the absorbance at 420nm during 10 min after the addition of *Bp*BOD (blue), *Bp*BOD/TCEP (orange), *Bp*BOD/TCEP/PyMal (green) and *Bp*BOD/PyMal (red) mixture into ABTS solution.

2. Supplementary Tables

Table S1. Estimation of the *Bp*BOD protein loading on the electrode surface.

	GCE/MWCNTs/ *Bp*BOD	GCE/MWCNTs/ TCEP-*Bp*BOD	GCE/MWCNTs/ TCEP-Py-Mal-*Bp*BOD, mix	GCE/MWCNTs/ TCEP-PyMa-*Bp*BOD, layers	GCE/MWCNTs/ *Bp*BOD - PyMal (without TCEP) mix	GCE/MWCNTs/ *Bp*BOD - Py-Mal (without TCEP) layers
**Average absorbance at 420nm**	0.074	0.061	0.164	0.091	0.076	0.085
**Amount of *Bp*BOD deposited on the electrode surface [μg]**	1.12	0.92	2.49	1.38	1.15	1.29

Table S2. Electron transfer rate (Ket) value estimation

	GCE/MWCNTs/ *Bp*BOD	GC/MWCNTs/ TCEP-*Bp*BOD	GC/MWCNTs/ TCEP-PyMal-*Bp*BOD , mix	GC/MWCNTs/ TCEP- PyMal - *Bp*BOD , layers	GCE/MWCNTs/ *Bp*BOD / Py-Mal without TCEP mix	GCE/MWCNTs/ *Bp*BOD / PyMal without TCEP layers
**Ket value [sec^{-1}]**	12.86	1.44	2.96	8.25	1.66	5.65
